# Developments and applications of α-bromonitrostyrenes in organic syntheses

**DOI:** 10.1039/d4ra02474e

**Published:** 2024-05-08

**Authors:** Fatemeh Doraghi, Mohammad Mahdi Aghanour Ashtiani, Fatemeh Moradkhani, Bagher Larijani, Mohammad Mahdavi

**Affiliations:** a Endocrinology and Metabolism Research Center, Endocrinology and Metabolism Clinical Sciences Institute, Tehran University of Medical Sciences Tehran Iran momahdavi@tums.ac.ir

## Abstract

The presence of the bromo and nitro groups in the structure of α-bromonitrostyrene makes them highly reactive and versatile reagents in organic syntheses. α-Bromonitrostyrenes act as an effective dielectrophile in the reaction with various nucleophiles. In these reactions, the bromo and nitro groups behave as good leaving groups for the assembly of a diverse range of heterocyclic compounds, such as dihydrofurans, dihydropyranes, furans, pyrroles, pyrazoles, isooxazolines, spiropyrrolidines, *etc.* In the current review, we have focused on the transformations of α-bromonitrostyrenes under organocatalysis, metal catalysis, and base-catalysis systems as well as catalyst-free conditions, since 2010.

## Abstract 2

In this work, we have described the use of α-bromonitrostyrenes in the synthesis of a wide variety of carbocyclic and heterocyclic compounds under organocatalysis, metal catalysis, and base-catalysis systems as well as catalyst-free reactions.

## Graphical abstract

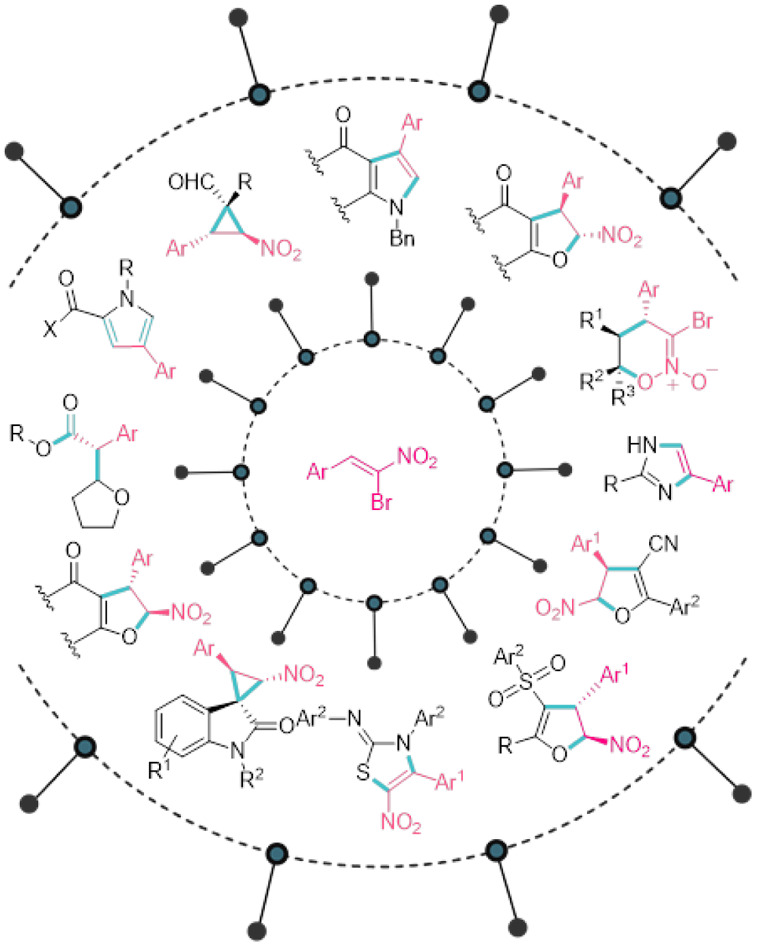

## 1. Introduction

**Scheme 1:**
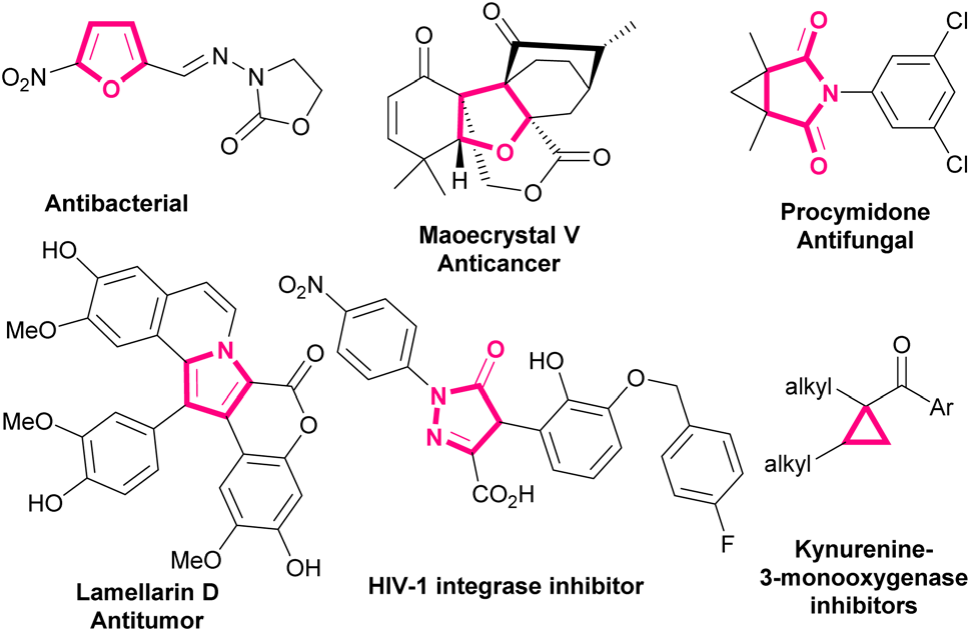
Selected examples of biologically active molecules containing pyrrole, furan, dihydrofuran, or cyclopropane rings.

Bromonitroalkenes, especially bromonitrostyrenes are versatile building blocks for the construction of biological and pharmaceutical molecules and optically active compounds, such as dihydrofurans,^1,2^ pyrroles,^3–6^ and pyrazoles.^7–9^ Selected examples of biologically active molecules containing pyrrole, furan, dihydrofuran, or cyclopropane cores are shown in Scheme 1.

Due to the presence of both nitro and bromo groups, α-bromonitrostyrenes exhibit peculiar properties in comparison with nitrostyrenes and bromostyrenes.^10–14^ These substituents can behave as good leaving groups in the nucleophilic substitution reactions. Because of this feature, α-bromonitrostyrenes can easily be used in Michael addition reactions as a dipolarophile to make various functionalized dihydrofurans, pyrroles, pyrazoles, bicyclic oximes, triazoles, imidazoles, cyclopropanes, *etc.*^15^ In such reactions, α-bromonitroalkenes act as a 1,2-bielectrophile in a diverse range of cascade reactions with binucleophiles, such as 1,3-dicarbonyl compounds, enamines, aldehydes, alkenes, phenols, diazo compounds and other miscellaneous reagents.

Michael addition reactions of α-bromonitroalkenes can be performed in the presence of organocatalysts, metal catalysts, and bases, as well as catalyst-free systems. Among them, advances in enantioselective organocatalytic Michael reactions have provided various enantiomerically enriched compounds and biologically active molecules.^16–19^ On the other hand, domino Michael/Henry reactions are known as powerful methods for rendering complex molecular architectures through constructing the carbon–carbon bonds in a one-pot procedure without isolating intermediates.^20,21^ Recently, progress in asymmetric domino reactions utilizing chiral organocatalysts has been well revealed and interesting achievements has been gained.^22–24^

Recently, various research teams, such as Jakubec,^25^ Gao,^26^ Lee,^27^ Halimehjani,^28^ Shen,^29^ and Zhang^30^ reported the organic transformations of nitrostyrenes. In this review, we have specifically discussed the use of α-bromonitrostyrenes in various types of catalytic systems, such as organocatalysis, metal catalysis and base catalysis as well as catalyst-free reactions. We have also highlighted important features of the reactions and mechanisms to familiarize the readers with the reactivity and behavior of α-bromonitrostyrenes in organic syntheses.

## 2. Transformations of bromonitrostyrenes

### 2.1. Organocatalyst-catalyzed transformations of bromonitrostyrenes

**Scheme 2:**
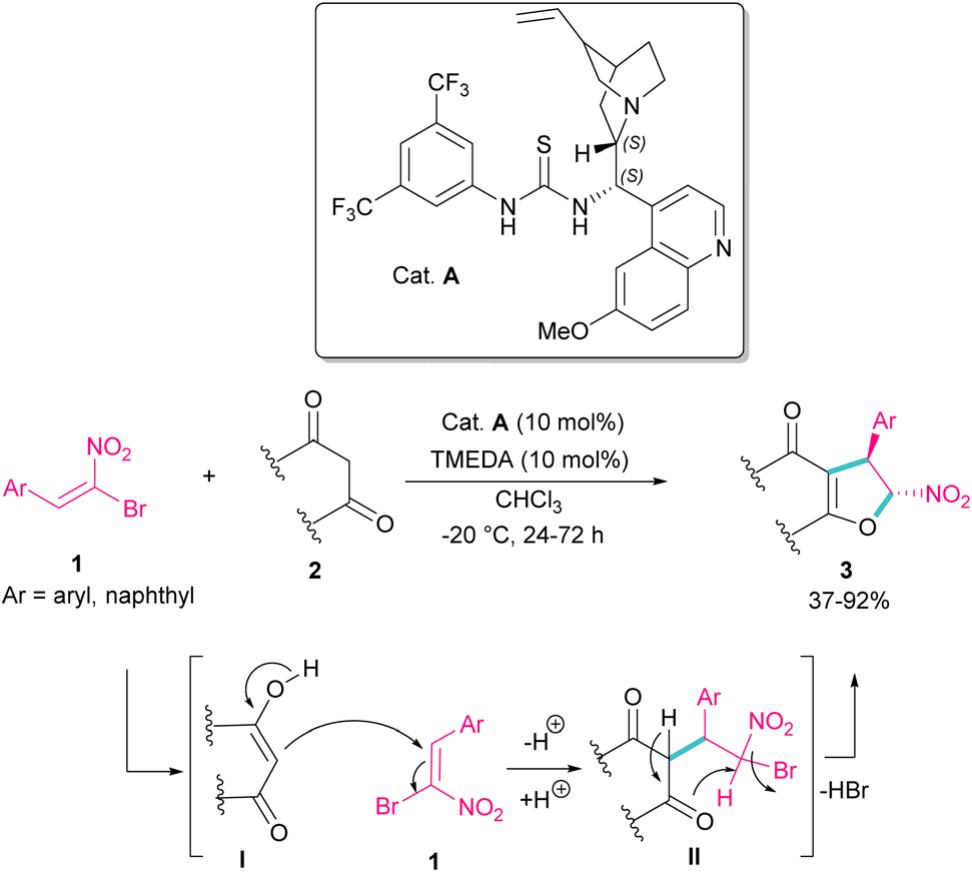
Organocatalysis reaction of (*E*)-β-bromonitrostyrenes with 1,3-dicarbonyl compounds.

In 2010, Rueping *et al.* used (*E*)-β-bromonitrostyrenes 1 for the synthesis of dihydrofurans 3 from 1,3-dicarbonyl compounds 2 (Scheme 2).^31^ In this reaction, the chiral thiourea catalyst A can close two substrates together by hydrogen-bonding interaction and facilitate Michael addition of diketone 2 as a dinucleophile to bromonitrostyrene 1 as an electrophile to generate intermediate II. Then, an intramolecular nucleophilic addition delivered product 3 by the release of HBr. The same organocatalyst was used in the reaction of bromonitrostyrene, as a dielectrophile and 1,3-indandione as a dinucleophile to make spiro-nitrocyclopropane in 57% yield with 35% ee.^32^

**Scheme 3:**
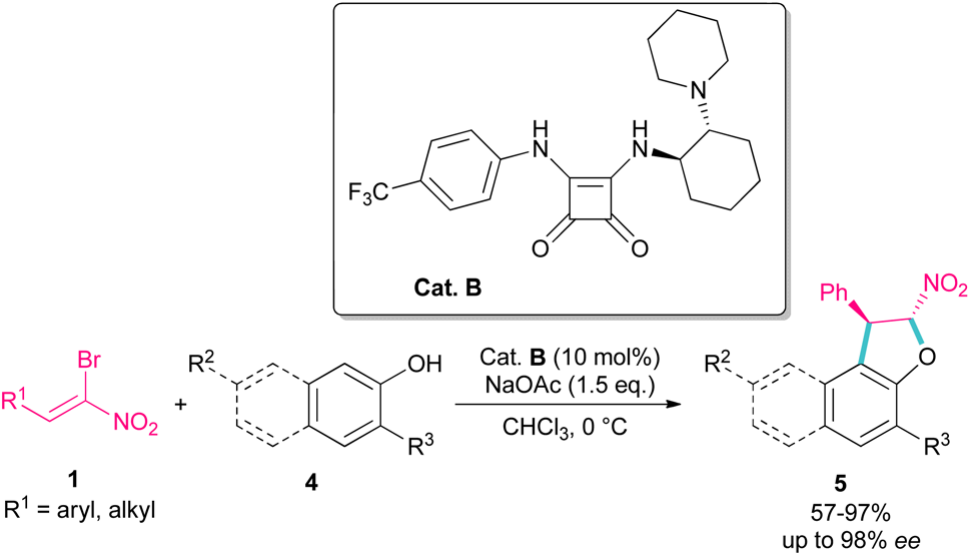
Reaction of (*Z*)-bromonitroalkenes and naphthols catalyzed by a chiral organocatalyst.

Parra and co-workers used an organocatalysis system to synthesize enantioselective dihydroarylfuran derivatives 5 from the cyclization reaction between (*Z*)-bromonitroalkenes 1 and naphthol 4 (Scheme 3).^33^ The hydrogen bonding interaction of chiral organocatalyst B with both substrates was responsible for the suitable orientation for Michael–Friedel–Crafts reaction and subsequent S_N_2 reaction on the carbon attached bromide. It should be noted that HBr resulting from the reaction can be neutralized with a stoichiometric amount of a base.

**Scheme 4:**
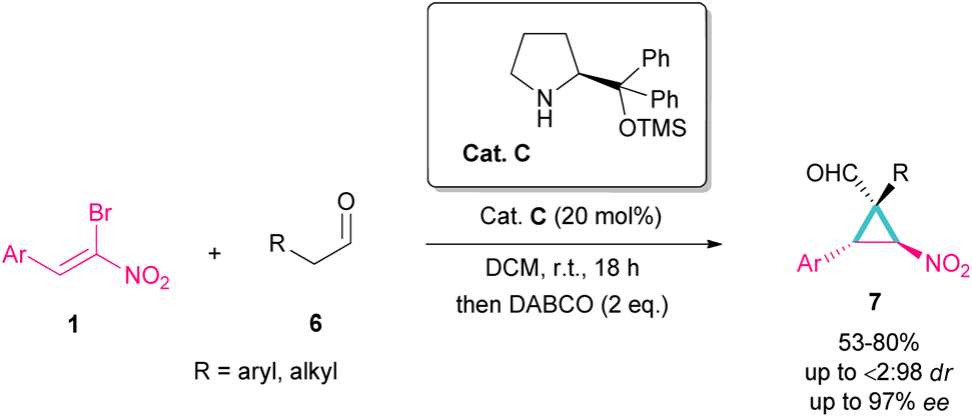
Reaction of bromonitroalkenes and alkyl aldehydes catalyzed by chiral organocatalyst.

**Scheme 5:**
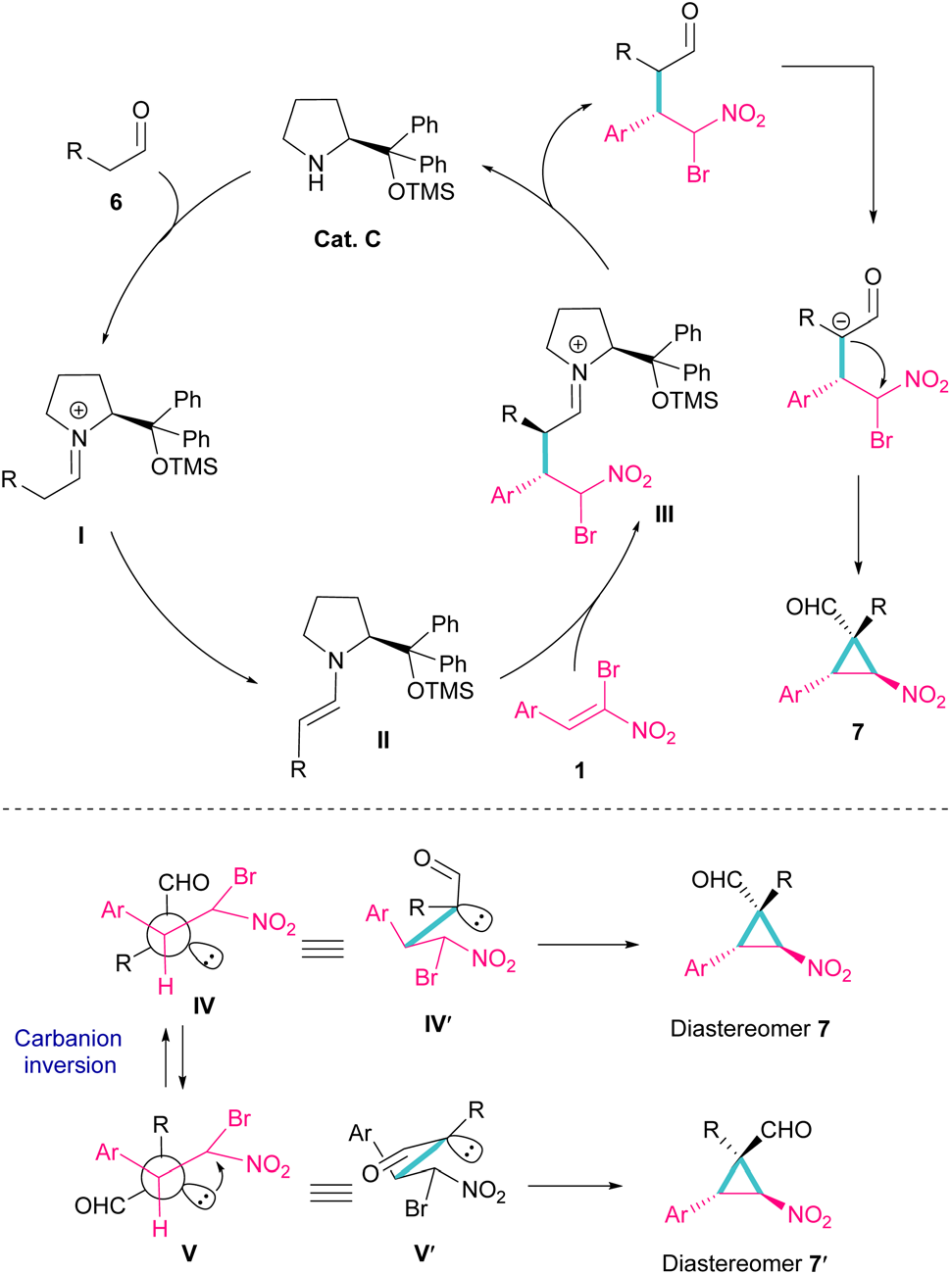
Putative mechanism for reaction of bromonitroalkenes and alkyl aldehydes.

Another organocatalyst was used in the reaction of bromonitroalkenes 1 and alkyl aldehydes 6 (Scheme 4).^34^ A series of enantioenriched cyclopropanes 7 with quaternary carbon centers were prepared in the presence of a chiral pyrrolidine catalyst C. As illustrated in Scheme 5, the reaction started with the condensation of pyrrolidine with aldehyde to form imine I, followed by a 1,3-H shift to enamine II. The attack of bromonitrostyrene to the alkene moiety produced intermediate III. By release of the catalyst and subsequent intramolecular S_N_2 reaction, cyclopropane 7 was provided along with the elimination of bromide. It seems that the formation of two diastereomers 7 and 7′ from two configurations IV and V is possible. The configuration at C2 is generated from the intramolecular attack of the anion with DABCO, while the configuration at C4 is from the acidic proton at this C-atom, providing the thermodynamically stable product. In this case, the NO_2_ group is at the *trans* position to the aryl ring at C3.

**Scheme 6:**
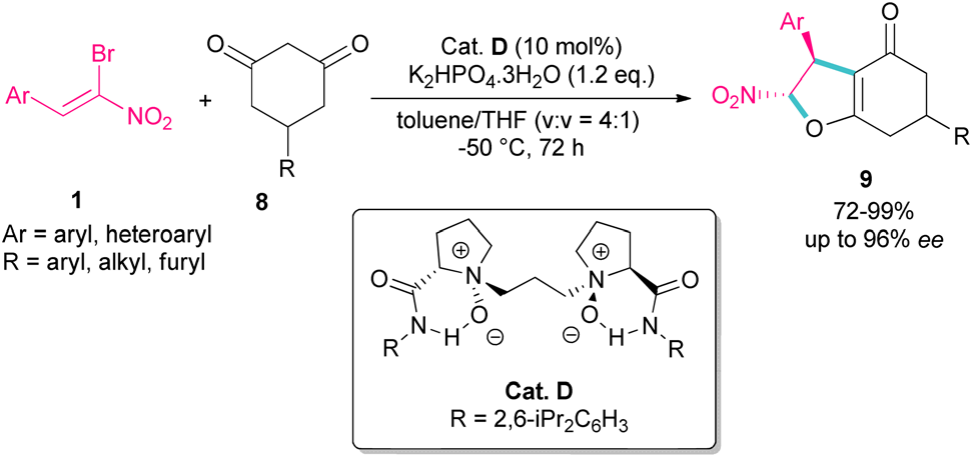
Reaction of cyclohexane-1,3-dione and bromonitrostyrenes catalyzed by organocatalyst.

A domino Michael-alkylation reaction was reported by Feng and co-workers for the synthesis of dihydrofurans 9 from bromonitrostyrene 1 (Scheme 6).^35^ For this purpose, they treated bromonitrostyrene 1 and cyclohexane-1,3-diones 8 in the presence of the organocatalyst D. For dimedone, two stereocenters of the bicyclic 2,3-dihydrofurans were obtained with high diastereo- and enantioselectivity, while three stereocenters of the bicyclic 2,3-dihydrofurans were prepared from prochiral 5-monosubstituted cyclohexane-1,3-dione products *via* the desymmetrization reaction.

**Scheme 7:**
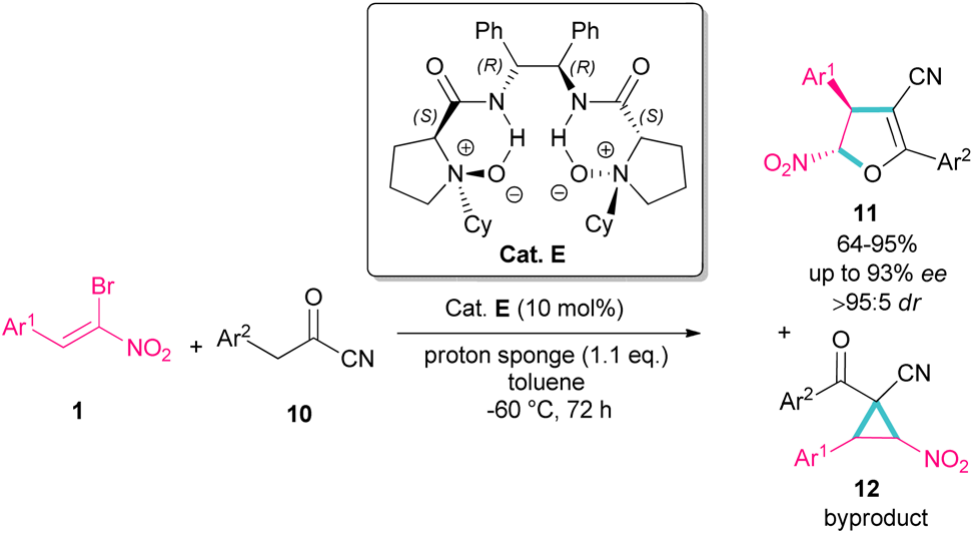
Organocatalysis reaction of (*Z*)-bromonitrostyrenes and α-substituted cyano ketones.

In 2016, Feng *et al.* introduced a chiral organocatalyst E for the asymmetric synthesis of dihydrofurans 11 from (*Z*)-bromonitrostyrenes 1 and α-substituted cyano ketones 10 (Scheme 7).^36^ In this regard, a domino Michael addition-alkylation between bromonitrostyrene and α-substituted cyano ketone in the presence of chiral *N*,*N*′-dioxide E was conducted under very mild reaction conditions. The authors found that the use of proton sponge, added in six equal parts, could enhance enantioselectivity and also the performance of the reaction in a very low temperature prevent the formation of the cyclopropane compound as a byproduct and thus increasing the yield of dihydrofuran 11. In addition to excellent enantioselectivity, high diastereo- and regioselectivity were also observed in this reaction.

**Scheme 8:**
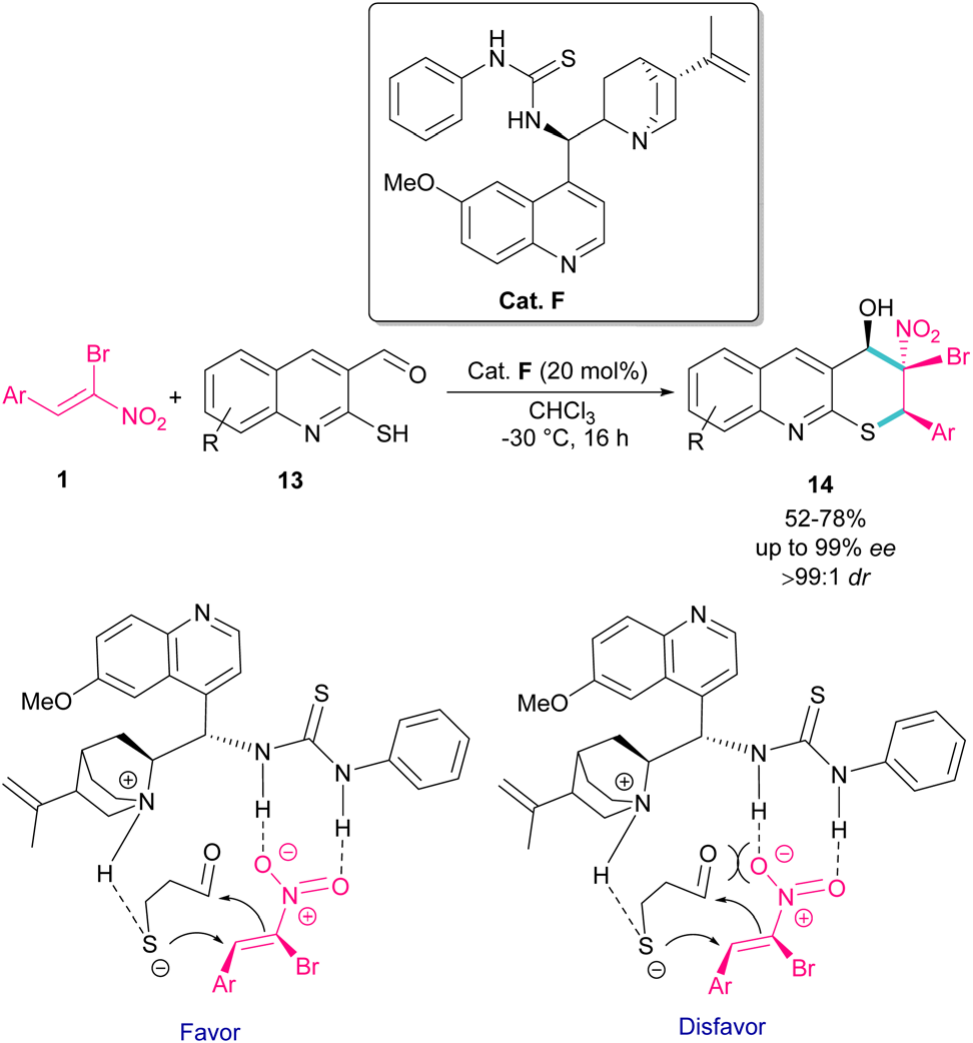
Organocatalyst-catalyzed reaction of 2-mercaptoquinoline-3-carbaldehyde and α-bromonitroalkene.

The use of α-bromonitroalkenes 1 in the synthesis of 3,4-dihydro-2*H*-thiopyrano[2,3-*b*]quinolines 14 was reported by Xie and co-workers in 2018 (Scheme 8).^37^ An organocatalysis system was proposed for this synthetic method, which started with the deprotonation of 2-mercaptoquinoline-3-carbaldehyde 13 by tertiary amine of catalyst F, and the activation of α-bromonitroalkene 1 by the thiourea moiety of F through the formation of two hydrogen bonds. The subsequent domino Michael/Henry reaction resulted in product 14. This method had the advantages of excellent enantio- and dieastereoselectivity, mild reaction conditions, and the ability of 3,4-dihydro-2*H*-thiopyrano[2,3-*b*]quinolines to undergo further organic transformations. In addition, salicylaldehyde can also act as a coupling partner in the cyclization with bromonitroalkene.

**Scheme 9:**
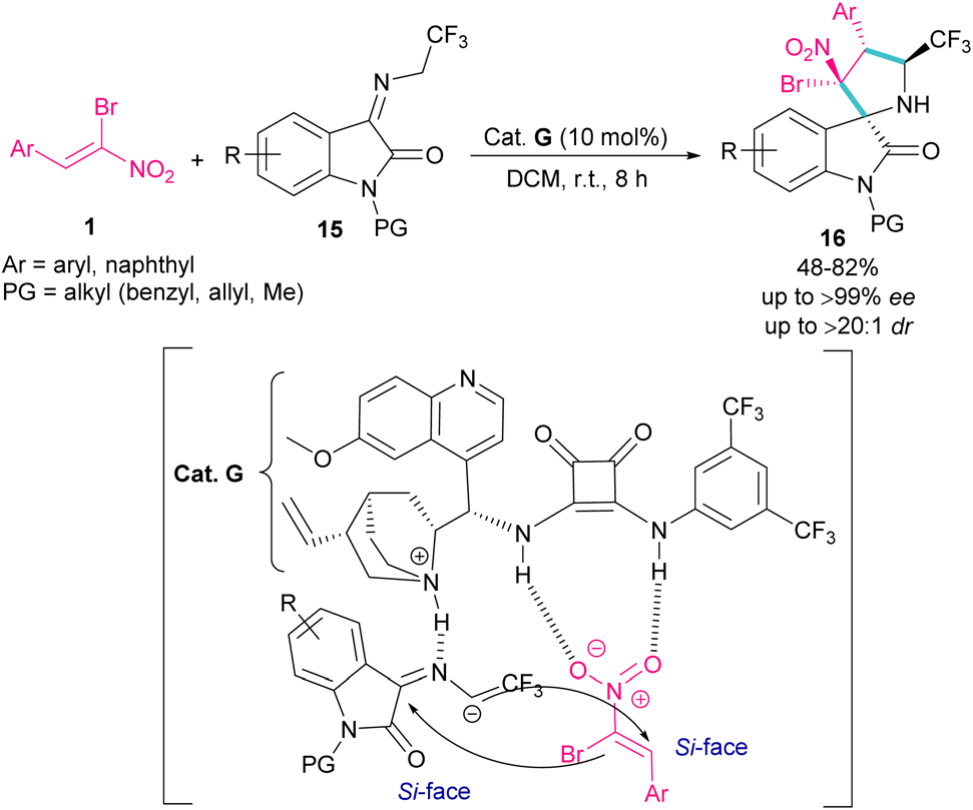
Reaction of isatin-derived ketimine and (*Z*)-α-bromonitroalkene catalyzed by cinchonidine-derived squaramide.

In 2020, an organocatalysis system was developed to prepare pyrrolidinyl spiro-oxindoles bearing quaternary carbon centers 16 (Scheme 9).^38^ Han and co-workers designed a (3 + 2)-cycloaddition reaction between isatin-derived ketimine 15 and (*Z*)-α-bromonitroalkene 1 in the presence of cinchonidine-derived squaramide G. This bifunctional organocatalyst can control the stereoselectivity by the hydrogen bonding interactions with both substrates blocking one side of each substrate, allowing nucleophilic attack from the *Si*-face.

**Scheme 10:**
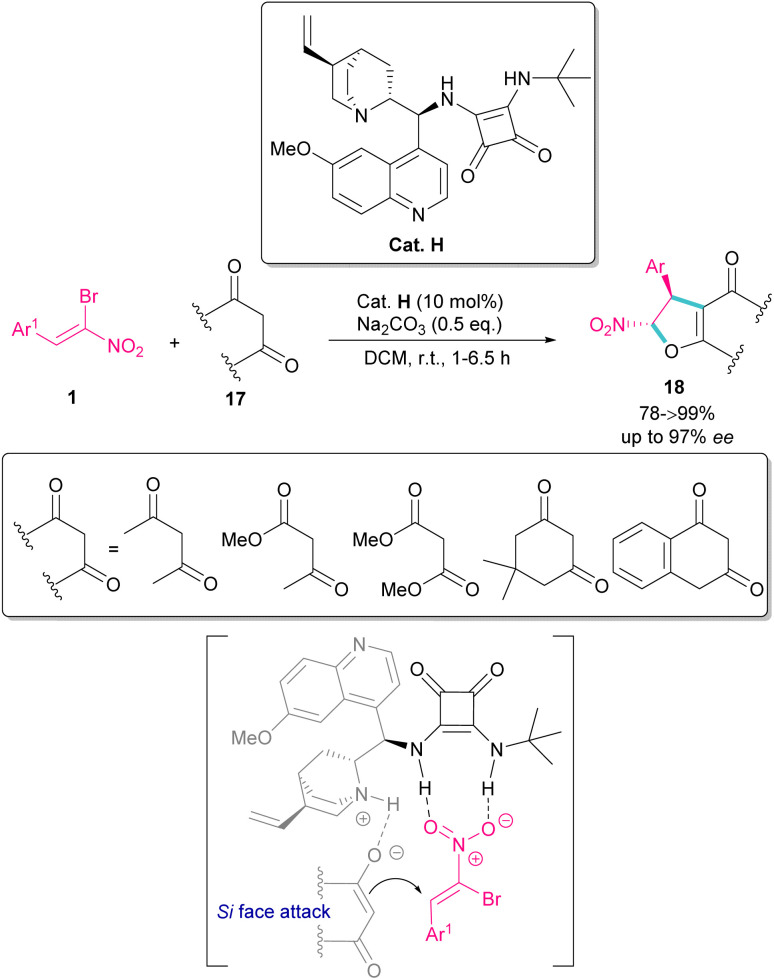
Squaramide-catalyzed reaction of bromonitroalkenes and 1,3-dicarbonyl compounds.

In 2022, Tanyeli and co-workers extended a new organocatalysis system for the synthesis of chiral dihydrofurans 18 from bromonitroalkenes 1 and 1,3-dicarbonyl compounds 17 (Scheme 10).^39^ Their reaction proceeded through domino type Michael-S_N_2 reactions in the presence of a quinine-derived sterically encumbered squaramide H. The main advantages of this method that differentiates it from previous methods was the performance of the reaction in room temperature at short reaction times, while other similar reactions required cryogenic conditions and longer times to proceed. As shown in transition state, the quinoline moiety of the catalyst H activates the nucleophile 17 by hydrogen bonding. At the same time, the electrophile 1 is also activated by double hydrogen bonding with the squaramide group of H. Consequently, the nucleophilic attack of 17 from the *Si*-face of 1 afforded product with excellent enantioselectivity.

### 2.2. Metal-catalyzed transformations of bromonitrostyrenes

**Scheme 11:**
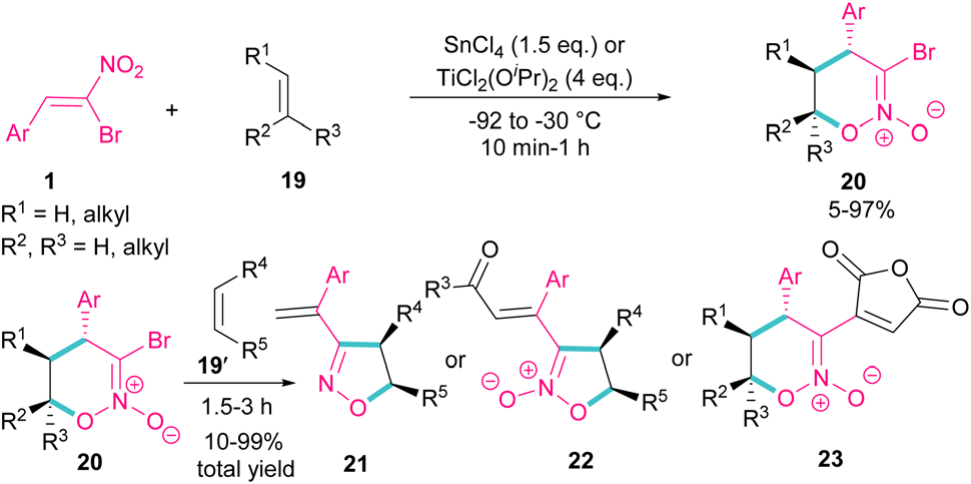
Reaction of bromonitrostyrenes with alkenes in the presence of SnCl_4_.

Bromonitrostyrenes can be applied for the construction of 3-bromo-5,6-dihydro-4*H*-1,2-oxazine *N*-oxides (Scheme 11).^40^ An alkene was used as a coupling partner and the cyclization reaction was performed in the presence of SnCl_4_ or TiCl_2_(O^*i*^Pr)_2_ as a Lewis acid catalyst. (4 + 2)-Cycloaddition of alkene to bromonitrostyrene was sensitive to the reaction conditions, where an increase in temperature or time led to 3-chloro-1,2-oxazine *N*-oxide as byproduct. In the next stage, (3 + 2)-cycloaddition of the obtained 3-bromo-5,6-dihydro-4*H*-1,2-oxazine *N*-oxides 20 with another alkene reactant 19′ was performed and three products, including 3-vinyloxazole 21, isoxazole *N*-oxide 22, and 3-functionalized 1,2-oxazine *N*-oxide 23 were obtained in different ratios.

**Scheme 12:**
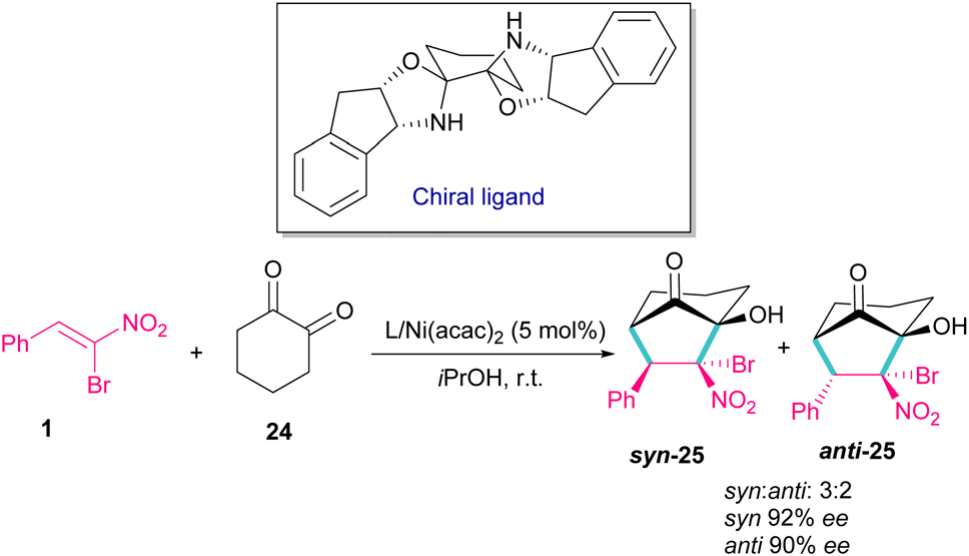
Ni-catalyzed reaction of bromonitrostyrenes with 1,2-cyclohexadione.

In 2014, a chiral bisoxazolidine in the combination with Ni(acac)_2_ catalyst was used for the preparation of optically active bicycle[3,2,1] octane of 1,2-diones 25 from nitroalkenes and 1 1,2-cyclohexadione 24 (Scheme 12).^41^ α-Bromonitroalkene 1 in a domino Michael–Henry reaction with diketone 24 can furnish product 25 with two *syn* and *anti* isomers. In this reaction, excellent enantioselectivity was observed (98% ee), but diastereoselectivity was relatively lower (2 : 1 dr) in comparison with other nitroalkenes as coupling reactants (up to 99% ee, up to 7 : 1 dr).

**Scheme 13:**
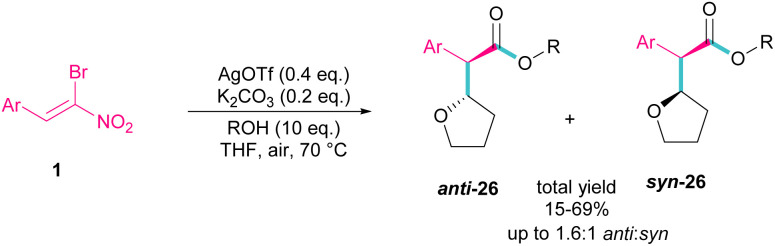
Ag-catalyzed addition of THF radicals to β-bromonitrostyrenes.

**Scheme 14:**
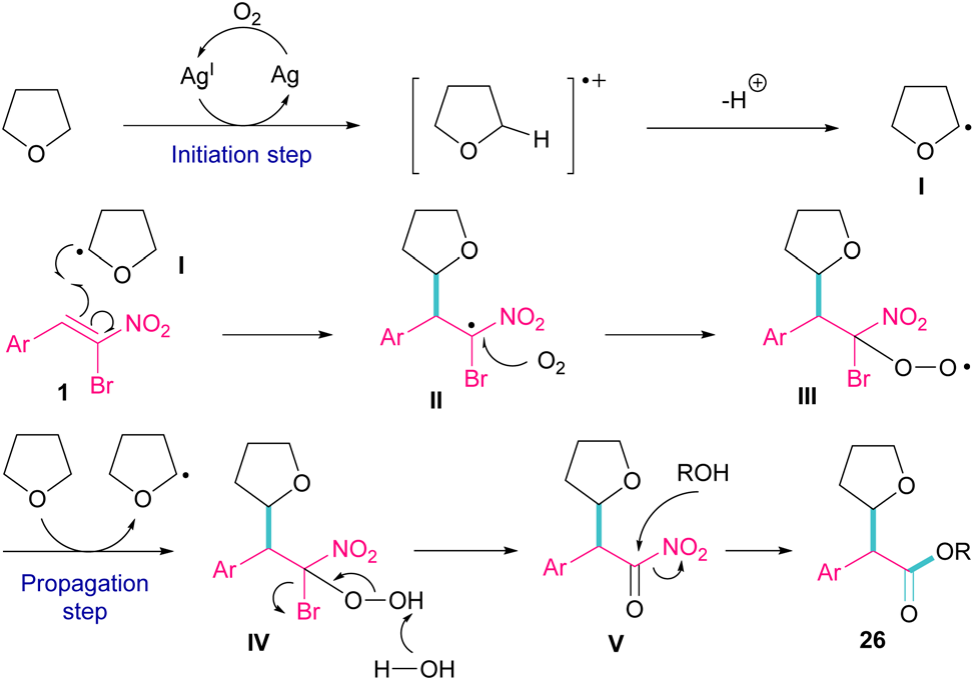
Plausible mechanism for Ag-catalyzed addition of THF radicals to β-bromonitrostyrenes.

Silver triflate can catalyze the three-component reaction of tetrahydrofuran, β-bromonitrostyrene and alcohol *via* a radical pathway (Scheme 13).^42^ The reaction was initiated by the formation of radical I from the interaction of Ag(i) with THF, which attacked β-bromonitrostyrene 1 to form intermediate II. Further attack of O_2_ on II led to the peroxy radical III. Then, III abstracted a hydrogen from THF to give hydroperoxide IV. The cleavage of the O–O bond by H_2_O resulted in intermediate V with the release of bromide. In this step, the nucleophilic attack of alcohol to the carbonyl group along with the removal of the second leaving group (NO_2_) led to product 26. It is noteworthy that only less hindered alcohols can attack intermediate V (Scheme 14).

### 2.3. Base-mediated transformations of bromonitrostyrenes

**Scheme 15:**
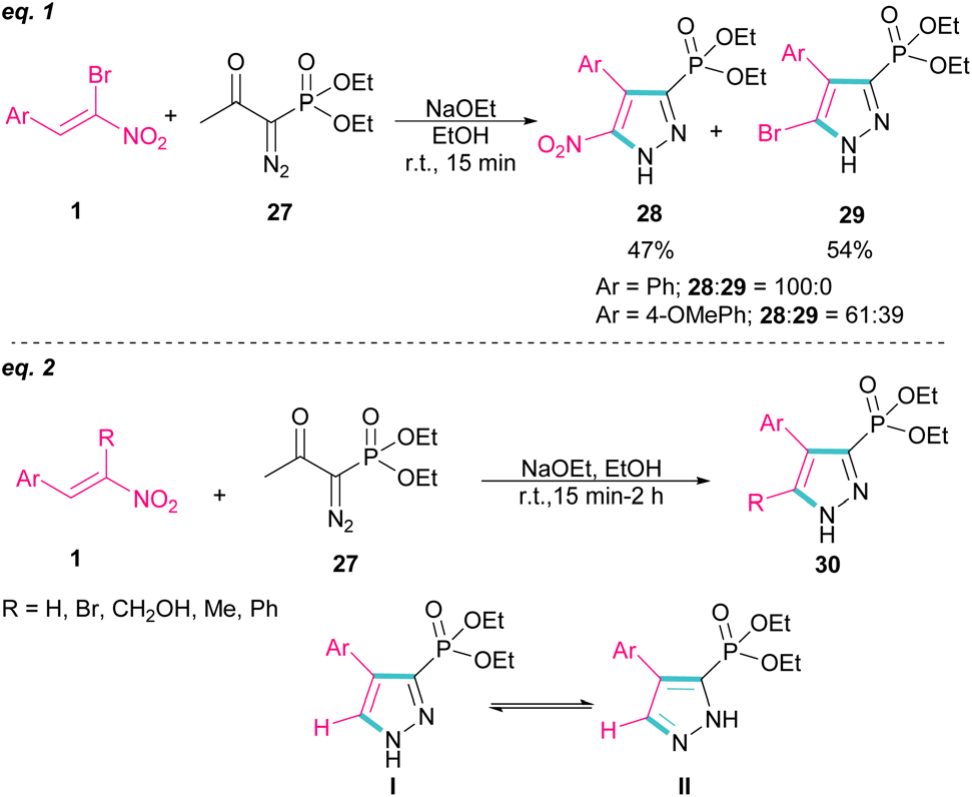
Reaction of diethyl 1-diazo-2-oxopropylphosphonate and α-bromonitroalkenes.

**Scheme 16:**
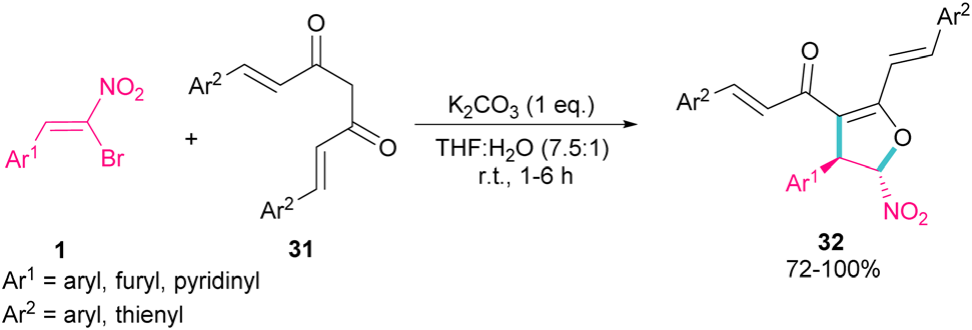
Base-mediated reaction of α-bromonitroalkenes with curcumins.

In 2007, Namboothiri and co-workers reported the synthesis of phosphonylpyrazoles 28 and 29 from diethyl 1-diazo-2-oxopropylphosphonate 27 with α-bromonitroalkenes 1 under basic conditions (Scheme 15, eqn (1)).^43^ The elimination of the leaving group in the final product depended on the substituent at the aryl ring of bromonitroalkene substrate. When Ar = Ph, product 28 was the major product, while products 28 and 29 were obtained in a ratio of 61 : 39 if bromonitrostyrene contained a 4-OMePh moiety. In a similar work, by dynamic NMR investigation, the Namboothiri group showed that two tautomers I and II are presented in such a reaction mixture that have a small energy difference but a high barrier to interconversion (Scheme 15, eqn (2)).^44^ Also, they could extend the substrate scope of bromonitrostyrenes in this work. Namboothiri *et al.* also reported the reaction of α-bromonitroalkenes 1 with curcumins 31 in the presence of a base (Scheme 16).^45^ Single diastereomer of dihydrofurans 32 was obtained in a Michael addition–alkenylation cascade reaction.

**Scheme 17:**
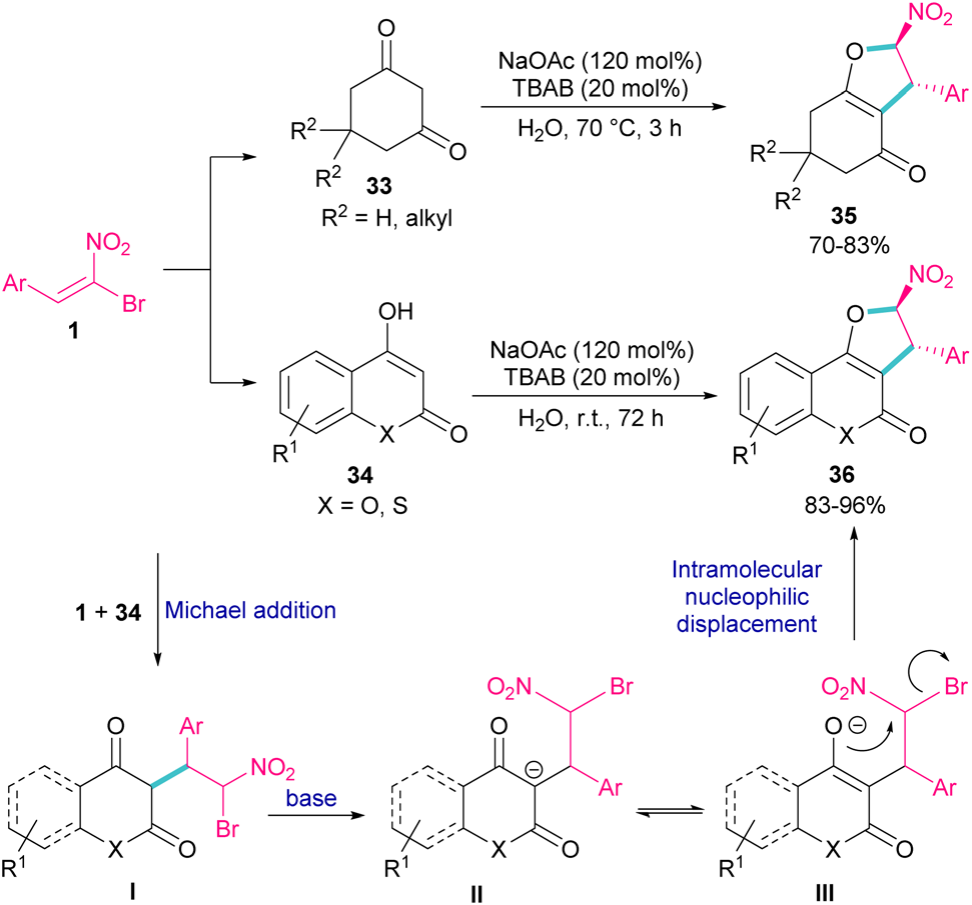
Reaction of bromonitrostyrenes with 4-hydroxycoumarins mediated by base.

**Scheme 18:**
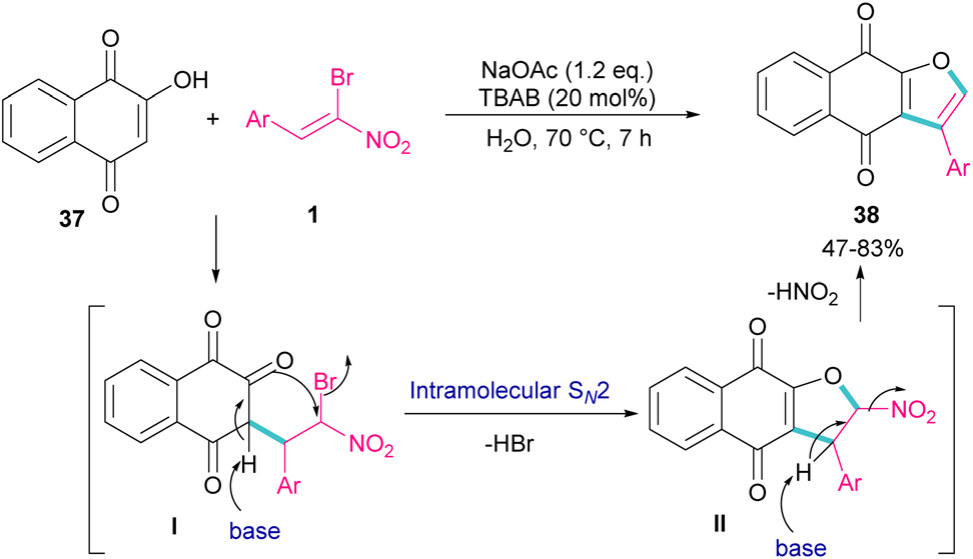
Reaction of α-bromonitroalkenes and 2-hydroxynaphthalene-1,4-diones under basic conditions.

In 2011, Xie *et al.* developed a method for the assembly of dihydrofurans from domino reaction of bromonitrostyrenes with 4-hydroxycoumarins or 1,3-carbonyl compounds (Scheme 17).^46^ In this work, tricyclic 2,3-dihydrofurans 36 and bicyclic 2,3-dihydrofurans 35 were obtained in high yields under basic conditions in a aqueous solution. In all cases, only the *trans* isomer of products was isolated, showing excellent diastereoselectivity of this method. Regarding the mechanism, Michael addition of bromonitroalkene 1 to 1,3-dicarbonyl compounds 33 gave intermediate I, which was converted to the enolate II under basic conditions. Finally, product 36 was formed *via* an intramolecular nucleophilic displacement in III. Similar reaction conditions were used by Jianwu and co-workers for the synthesis of furan structures (Scheme 18).^47^ In their reaction, α-bromonitroalkenes 1 and 2-hydroxynaphthalene-1,4-diones 37 were applied as starting materials to form 3-phenylnaphtho[2,3-*b*]furan-4,9-diones 38*via* Michael addition and subsequent intramolecular S_N_2 reaction. At last, the absorption properties of the obtained products were determined by UV-Vis spectra and fluorescence spectroscopy.

**Scheme 19:**
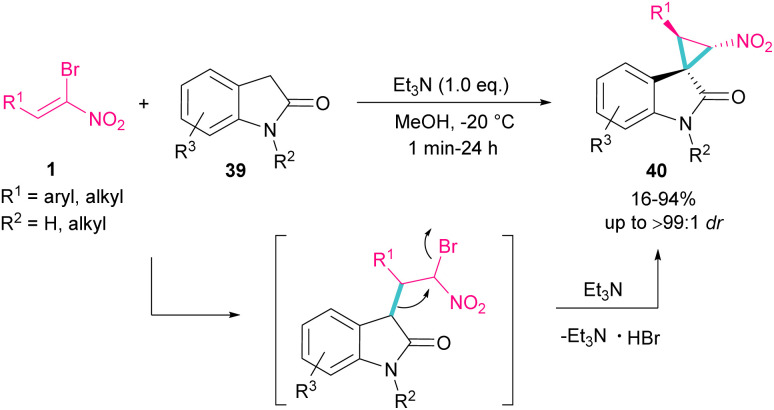
Reaction of bromonitroalkene with *N*-protected indolin-2-ones.

Spirocyclopropyl oxindole frameworks 40 were constructed in high yield with excellent diastereoselectivity from the reaction of bromonitroalkene 1 and *N*-protected indolin-2-ones 39 (Scheme 19).^48^ Due to high reactivity of indolin-2-one under ambient temperature and consequently low diastereoselectivity of the obtained product, a very low temperature was necessary for this reaction. The formation of cyclopropane was accomplished by the abstraction of a proton by a base and the liberation of bromide. Not only *N*-protected indolin-2-one but also *N*-H indolin-2-one was included in this transformation.

**Scheme 20:**
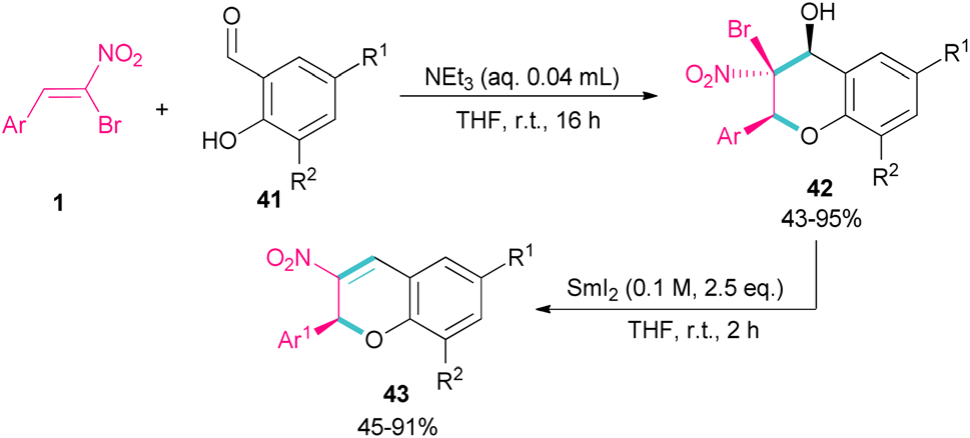
NEt_3_-catalyzed reaction of bromonitroalkene with *ortho*-hydroxybenzaldehydes.

Soengas *et al.* established a two-step procedure for the preparation of 2-*C*-glycosyl-3-nitrochromenes 43 starting from bromonitrostyrenes 1 and *ortho*-hydroxybenzaldehydes 41 (Scheme 20).^49^ For this purpose, first, they treated bromonitrostyrenes 1 with *ortho*-hydroxybenzaldehydes 41 under basic conditions to achieve (2*S*,3*S*,4*S*)-3-bromo-3,4-dihydro-4-hydroxy-3-nitro-2*H*-1-benzopyrans 42 in moderate to excellent yields. In the next stage, the reaction was conducted in the presence of 0.1 M solution of SmI_2_ in THF for couple hours to form 3-nitrochromenes 43. SmI_2_ can promote the β-elimination in benzopyran 42 with complete stereoselectivity. All steps were performed under mild conditions and all products were obtained with enantiomeric purity.

**Scheme 21:**
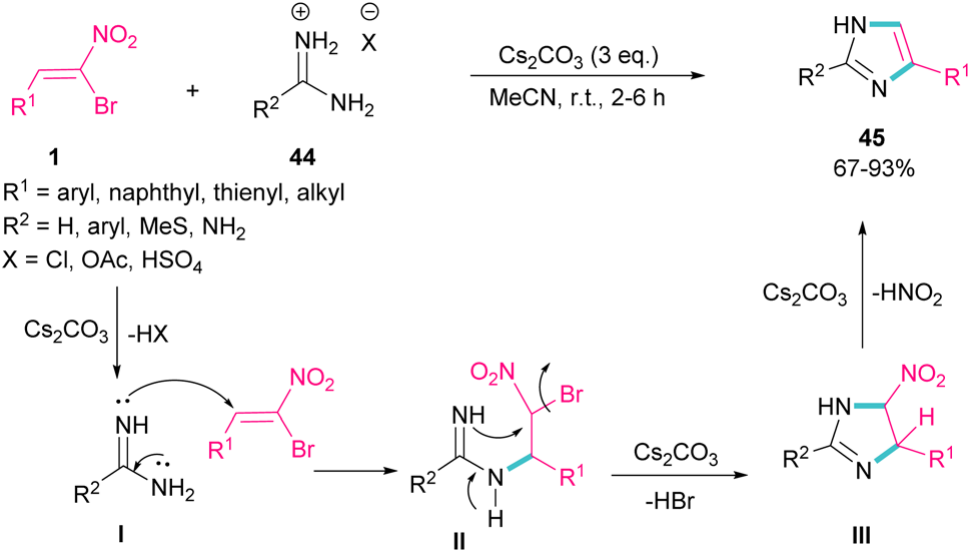
Cs_2_CO_3_-promoted reaction of bromonitroalkene with amidines.

In 2015, again the Namboothiri research team synthesized imidazole scaffolds 45 through the reaction of amidine hydrochloride 44 with α-bromonitroalkenes 1 (Scheme 21).^50^ For the assembly of 2,5-disubstituted imidazoles from α-bromonitroalkenes, 3.0 equivalents of Cs_2_CO_3_ were required to promote all steps of this imidazole synthesis. First, Cs_2_CO_3_ promoted the formation of amidine I by the elimination of HX form 44. Michael addition of 1 to I gave intermediate II, which underwent an intramolecular S_N_2 process *via* a 5-*exo-tet* fashion to provide nitroimidazoline III along with the removal of HBr in the presence of Cs_2_CO_3_. Subsequent elimination of HNO_2_ by Cs_2_CO_3_ led to product 45. Moreover, the authors reported potential anti-parasitic activity of the resulting imidazoles.

**Scheme 22:**
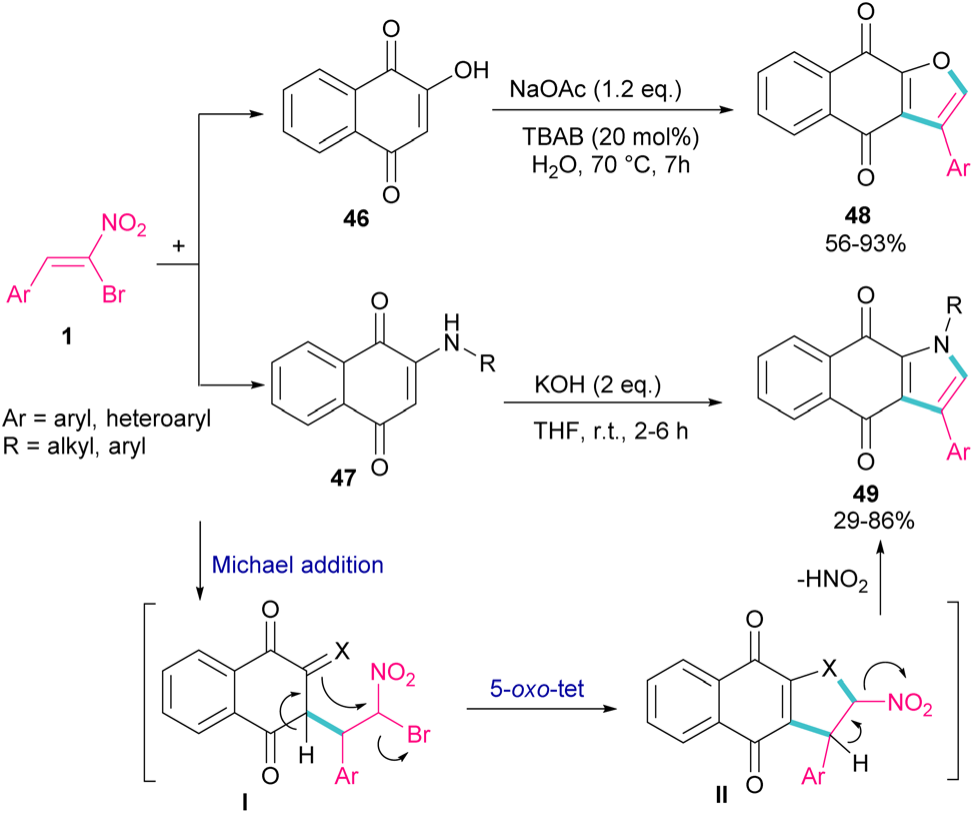
Reaction of bromonitroalkene with lawsone and 2-aminonaphthoquinone under basic conditions.

**Scheme 23:**
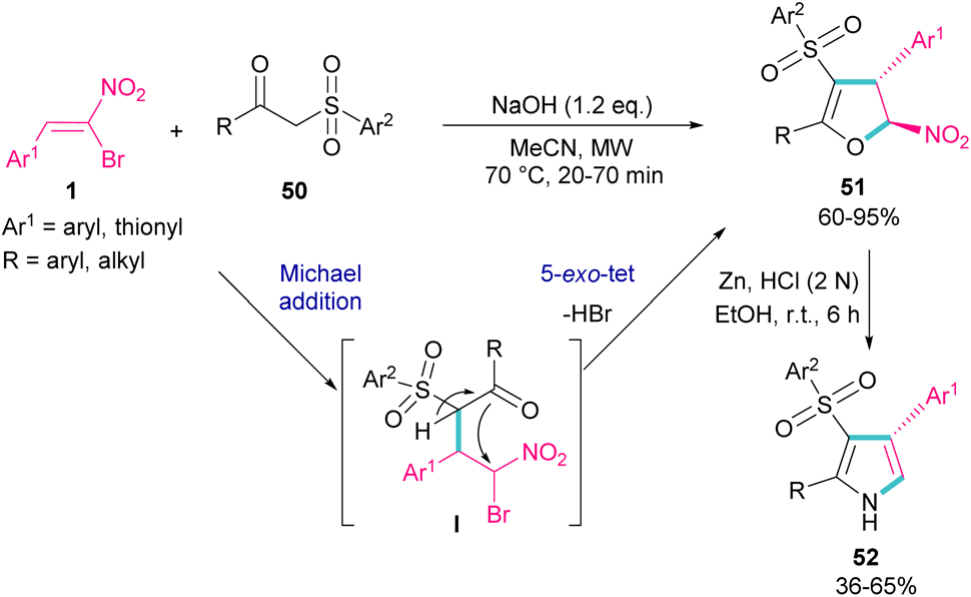
Reaction of bromonitroalkene with β-ketosulfones.

In 2018, Namboothiri and co-workers used α-bromonitroalkene 1 in the reaction with lawsone 46 and 2-aminonaphthoquinone 47 to produce furan and pyrrole fused quinonoid compounds 48 and 49 using two different basic conditions (Scheme 22).^51^ The transformations were performed *via* Michael addition of 1 to 47 to form intermediate I, followed by S_N_2 reaction in a 5-*exo-tet* manner to obtain intermediate II. Finally, pyrrole or furan frameworks were furnished after HNO_2_ removal. The authors also studied the anticancer activity of the obtained products. After a while, Namboothiri proposed a similar mechanism involving Michael addition/5-*exo-tet* reaction for the construction of dihydrofuran derivatives 51 from α-bromonitroalkenes 1 and β-ketosulfones 50 (Scheme 23).^52^ They could provide a series of pyrroles 52 by the reduction of dihydrofuran 51 in the presence of zinc powder under acidic conditions.

**Scheme 24:**
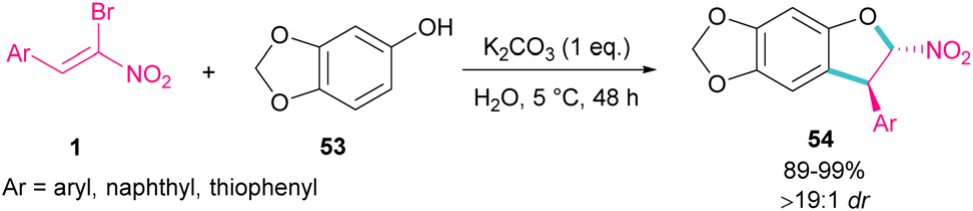
Reaction of (*Z*)-bromonitrostyrenes and sesamol in the presence of K_2_CO_3_.

In 2020, Feng *et al.* presented a protocol for the construction of diastereoselective *trans*-3-aryl-2-nitro-2,3-dihydrobenzofurans 54 under basic conditions (Scheme 24).^53^ A diverse range of (*Z*)-bromonitrostyrenes 1, containing aryl, naphthyl and thiophenyl moieties reacted smoothly with sesamol 53 in water as a green solvent to form dihydrofurans in excellent yields. All products were purified only by a simple filtration procedure and could also be synthesized in the Gram-scale (1.20 g, 94%). In the same year, the synthesis of the oxazole rings from α-halo-β-naphthol and nitroalkenes was carried out.^54^ Bromonitroalkene as a coupling partner gave the desired product in 82% with >20 : 1 diastereoselectivity.

**Scheme 25:**
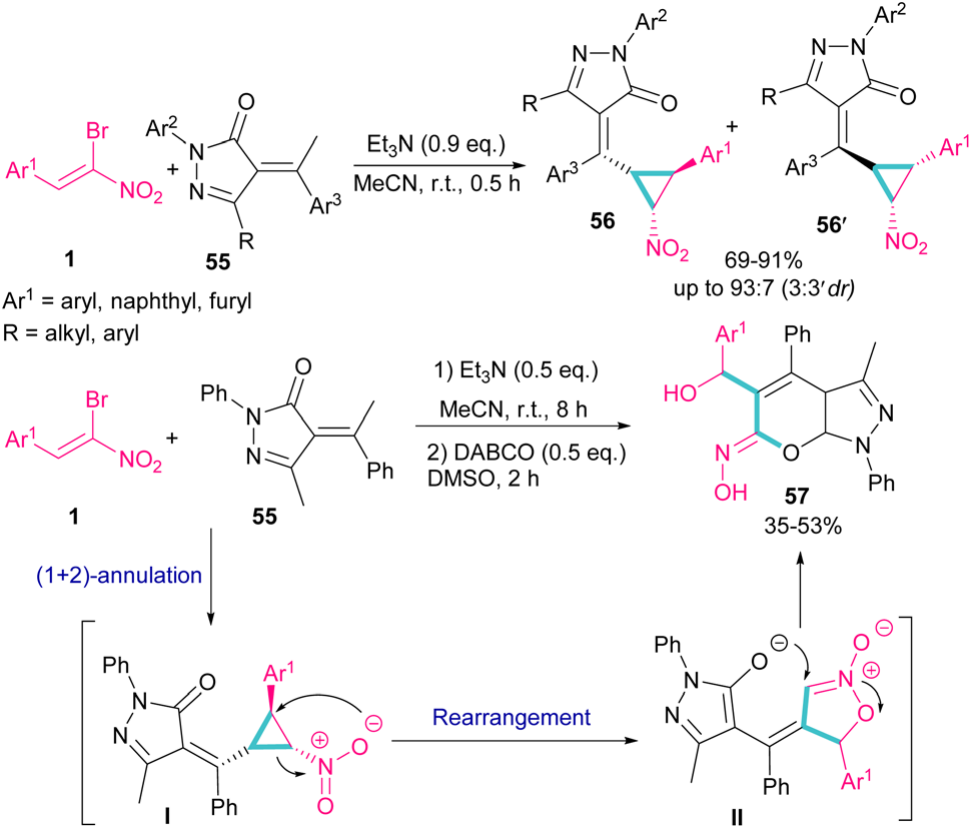
Reaction of bromonitrostyrenes with α-alkylidene pyrazolones mediate by Et_3_N.

A chemoselective annulation of bromonitrostyrenes with α-alkylidene pyrazolones was established by Han and co-workers in 2021 (Scheme 25).^55^ A novel library of pyrazole-fused pyranone oximes were obtained, where α-alkylidene pyrazolone 55 acted as C1 synthon and react in (2 + 1)-cycloaddition with bromonitrostyrene 1 to provide vinylcyclopropane-based pyrazolone 56 and 56′ in the presence of a base. The formation of diastereoisomer 56 was favored due to the less steric hindrance between the aryl and nitro groups. In another stage, the authors conducted this reaction in two steps, in which Et_3_N was served as a base to obtain the cyclopropane product. Then, the addition of second base resulted in an intramolecular rearrangement towards the synthesis of pyrazole-fused pyranone oximes 57.

**Scheme 26:**
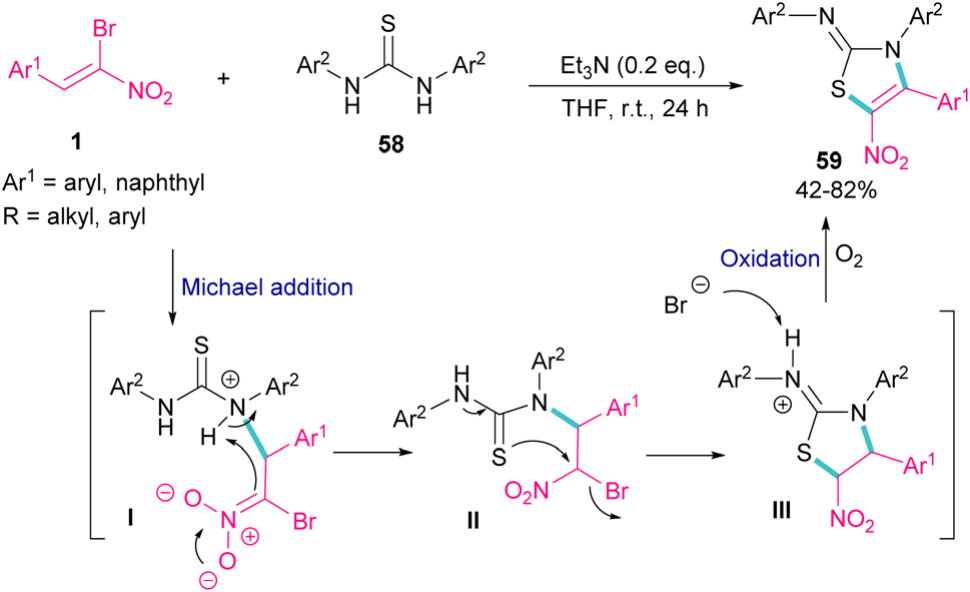
Et_3_N-promoted reaction of 1,3-disubstituted thioureas with 1-bromo-1-nitroalkenes.

The synthesis of 2-iminothiazolines 59 can be obtained from the cyclization of 1,3-disubstituted thioureas 58 with 1-bromo-1-nitroalkenes 1 (Scheme 26).^56^ At first, Michael addition between 1-bromo-1-nitroalkenes 1 and 1,3-diphenylthiourea 58 produced intermediate II, which then underwent a tautomerism and nucleophilic substitution to afford the five-membered ring intermediate III. The deprotonation of III by bromide anion, followed by the oxidation yielded product 59. It should be noted that using the suitable amount of the base and the performance of the reaction under air is crucial for this reaction to proceed.

**Scheme 27:**
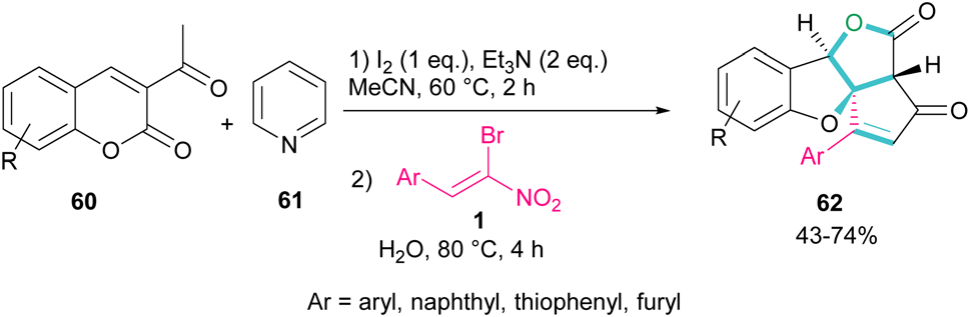
Three-component reaction of β-bromo-β-nitrostyrenes, 3-acetyl-2*H*-chromen-2-ones, and pyridine.

**Scheme 28:**
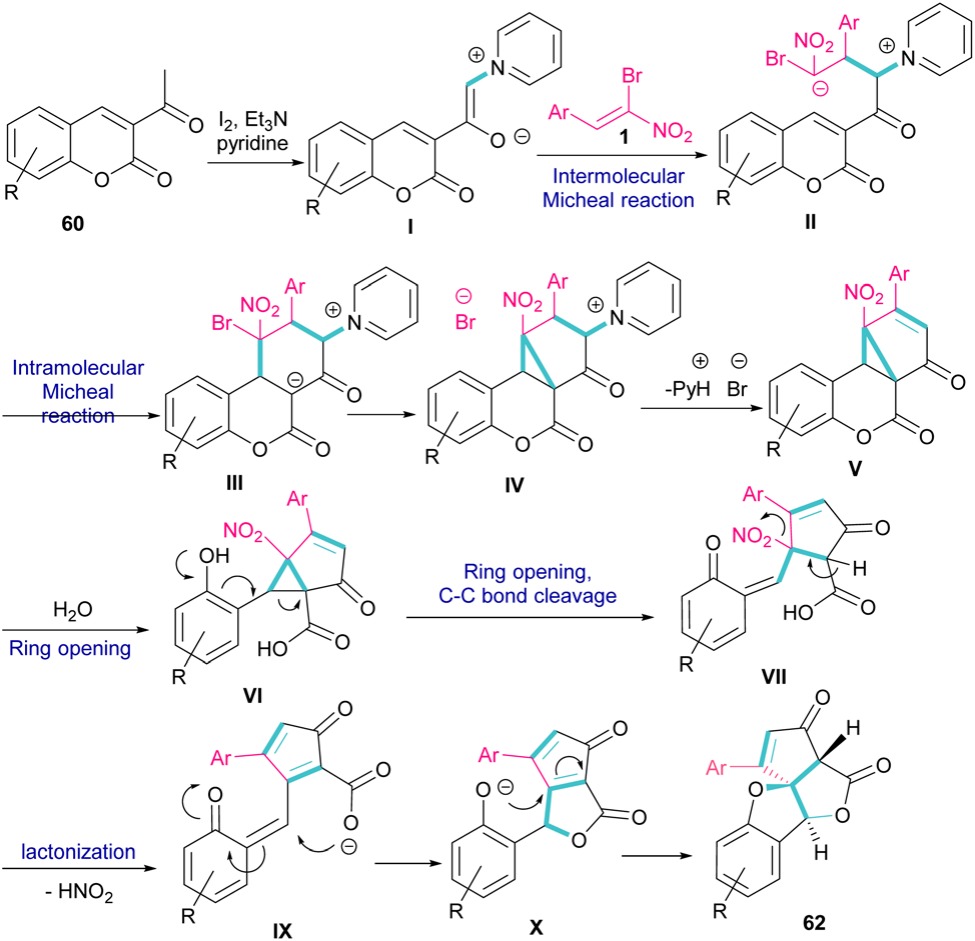
Possible mechanism for reaction of β-bromo-β-nitrostyrenes, 3-acetyl-2*H*-chromen-2-ones, and pyridine.

A new class of cyclopenta[*c*]furo[3,2-*b*]furan-5,6-diones, containing three carbon stereocenters were constructed by Yavari *et al.* in 2022 (Scheme 27).^57^ A three-component reaction, including β-bromo-β-nitrostyrenes 1, 3-acetyl-2*H*-chromen-2-ones 60, and pyridine 61 was carried out through three steps intermolecular and intramolecular Michael additions. Initially, the C–H bond functionalization of 3-acetyl-2*H*-chromen-2-one 60 with pyridine 61 in the presence of I_2_ and Et_3_N furnished pyridinium ylide I. Then, sequentially intermolecular Michael addition of 1 with I, and intramolecular Michael reaction gave intermediate II, which was converted to cyclopropane III. Intermediate IV was obtained *via* the elimination of pyridinium bromide in III, followed by two steps cyclopropane ring-opening and rearrangement to produce VI. The release of HNO_2_ gave VII, which underwent an intramolecular lactonization to render IX. Another intramolecular Michael addition of the phenoxide ion with the C=C bond gave product 62 (Scheme 28).

**Scheme 29:**
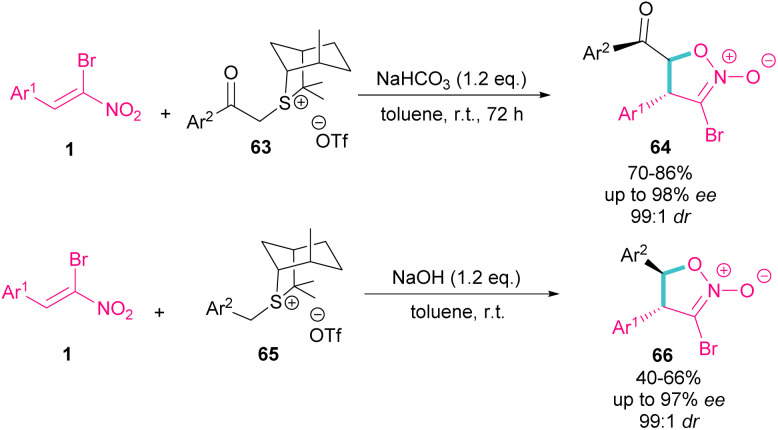
Reaction of sulfonium salts and substituted α-bromonitroalkenes.

In the same year, the Xie group designed a (4 + 1) ylide annulation between chiral sulfonium salts 63 and 65 with α-bromonitroalkenes 1 access to enantioenriched isoxazoline *N*-oxides 64 and 66 (Scheme 29).^58^ Two types of chiral sulfonium salts, such as α-benzoyl sulfonium triflate salts 63 and α-benzyl sulfonium triflate salts 65 could smoothly generate the sulfonium ylide intermediates, which served as C1 synthon in (4 + 1)-annulation with bromonitroalkene as a 4-atomic synthon. All products were obtained in high yields with excellent enantio- and diastereoselectivity. Also, changing the anions of sulfonium salts from OTf^−^ to Br^−^, ClO_4_^−^ and BF_4_^−^, showed that the type of anion has a great effect on the activity, and enantioselectivity in this asymmetric annulation.

**Scheme 30:**
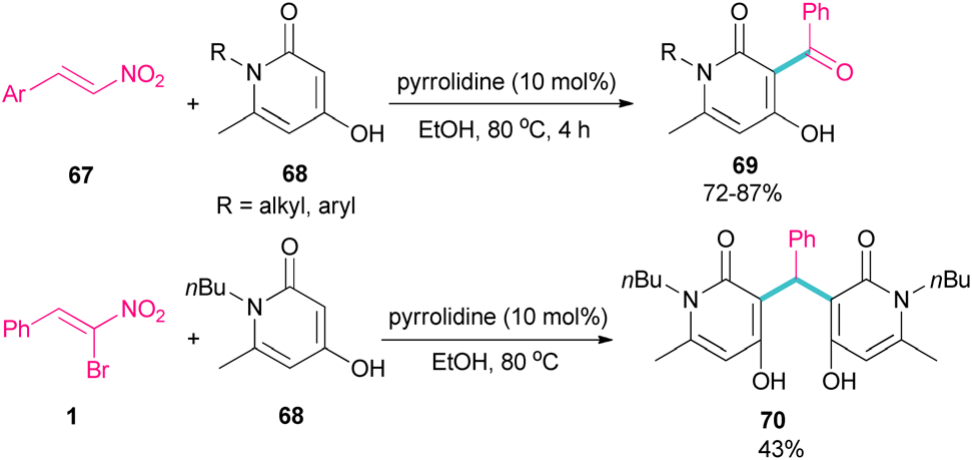
Pyrrolidine-catalyzed reaction of 1-butyl-4-hydroxy-6-methylpyridin-2(1*H*)-one and nitroalkenes.

In 2023, Li *et al.* investigated the application of pyrrolidine as a catalyst in the reaction of 1-butyl-4-hydroxy-6-methylpyridin-2(1*H*)-one 68 and nitroalkenes 67 (Scheme 30).^59^ They achieved an open ring product 69, when α-bromonitrostyrene 1 was used as a coupling reactant, while the reaction of other nitroalkenes led to 4-hydroxy-3-benzoylpyridin-2(1*H*)-ones 70 as the main product. This may be because of the high steric hindrance in α-bromonitrostyrene, which inhibits the formation of product 69.

### 2.4. Catalyst-free transformations of bromonitrostyrenes

**Scheme 31:**
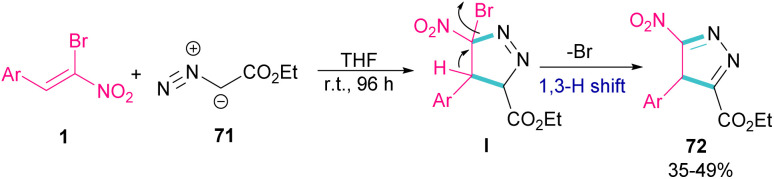
Reaction of α-bromo-α-nitroalkenes with ethyl diazoacetate under catalyst-free conditions.

In 2009, Xie and co-workers reported the synthesis of pyrazoles 72 from 1,3-cycloaddition of α-bromo-α-nitroalkenes 1 with ethyl diazoacetate 71 under catalyst-free conditions (Scheme 31).^60^ In general, the reaction involved the nucleophilic addition of 71 to 1 to form intermediate I, followed by the elimination of bromide along with the 1,3-H shift to furnish the final pyrazole product 72.

**Scheme 32:**
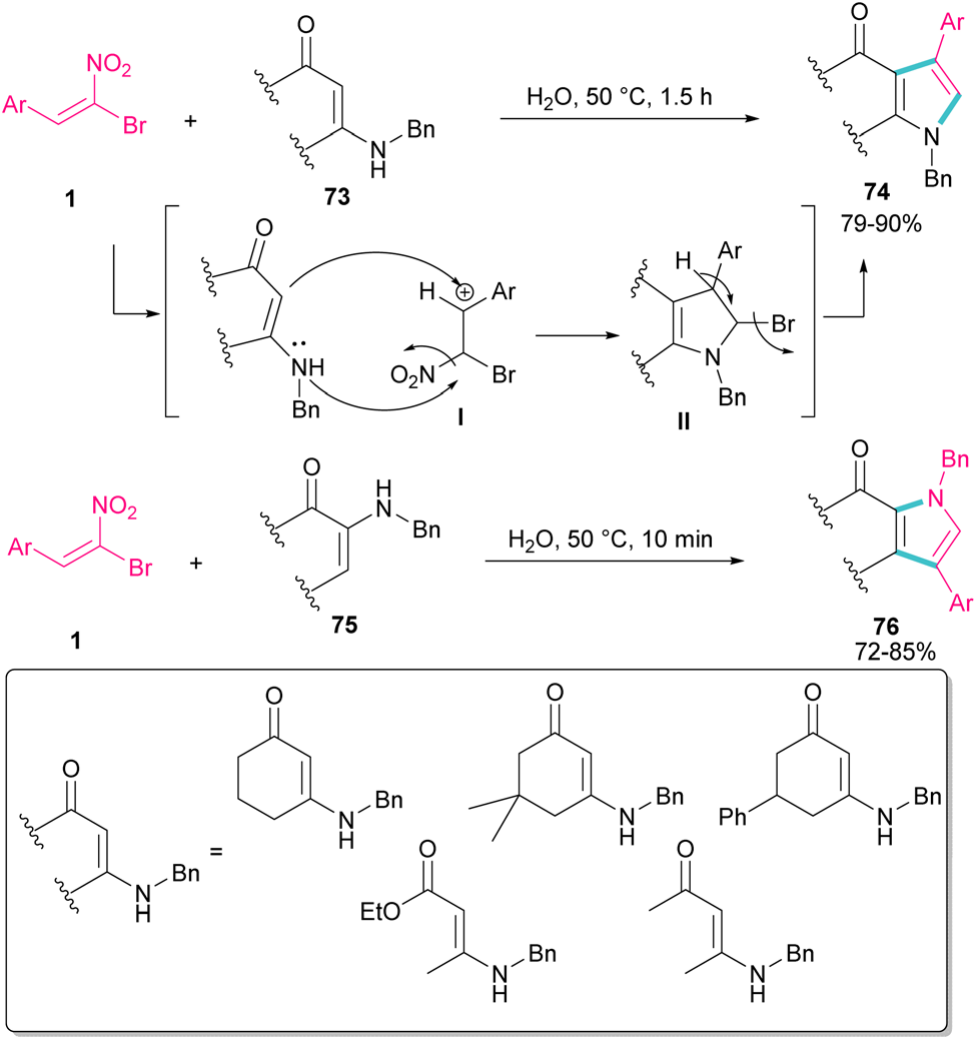
Catalyst-free reaction of (*E*)-β-bromonitrostyrenes with enaminones.

In 2010, Rueping and Parra treated (*E*)-β-bromonitrostyrenes 1 with enaminones 73 to synthesize pyrrole derivatives 74 (Scheme 32).^61^ The main advantages of their reaction were the performance of the reaction in water as a green solvent, very short reaction time, and excellent product yields. In this reaction, bromonitrostyrenes 1 acted as a trifunctional synthon and reacted well with both enaminone 73 and *N*-benzylenaminone 75 as binucleophilic synthons. First, the nucleophilic attack of nitrogen to the carbon atom in I concomitant with removal of a leaving group produced II. Next, the deprotonation and the release of another leaving group afforded the desired product 74.

**Scheme 33:**
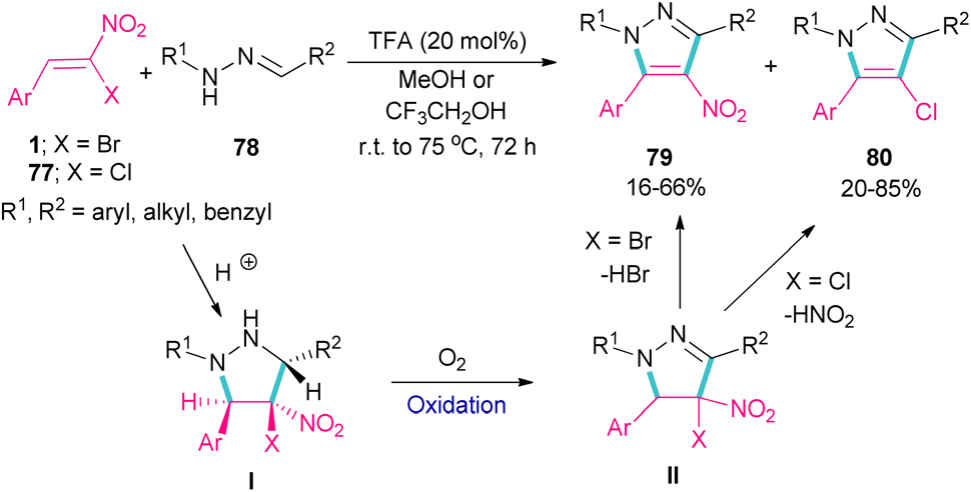
TFA-catalyzed reaction of bromonitrostyrenes with hydrazones.

Deng *et al.* showed that depending on the type of nitrostyrenes and the reaction solvent, two different pyrazole products could be obtained under acidic conditions (Scheme 33).^62^ When β-chloro-β-nitrostyrenes 77 were used as reactants in the reaction with hydrazones 78, the elimination of HNO_2_ occurred to produce 4-chloro-tetrasubstituted pyrazoles 80 in MeOH as a solvent. Whereas, 4-nitro-tetrasubstituted pyrazoles 79 were obtained from β-bromo-β-nitrostyrenes 1 and hydrazones 78 as starting materials in MeOH. In this case, the removal of HBr was favored over HNO_2_, which could be due to the easy cleavage of the C–Br bond compared to the C–Cl bond. In fact, the leaving-group abilities of functional groups can be classified as Br > NO_2_ > Cl. In general, the reactions involved pyrazolidine intermediate I, which was subjected to oxidation and elimination steps. It should be noted that when a more acidic alcoholic solvent like CF_3_CH_2_OH was utilized, the formation of 4-bromo-tetrasubstituted pyrazoles was also observed in the reaction mixture.

**Scheme 34:**
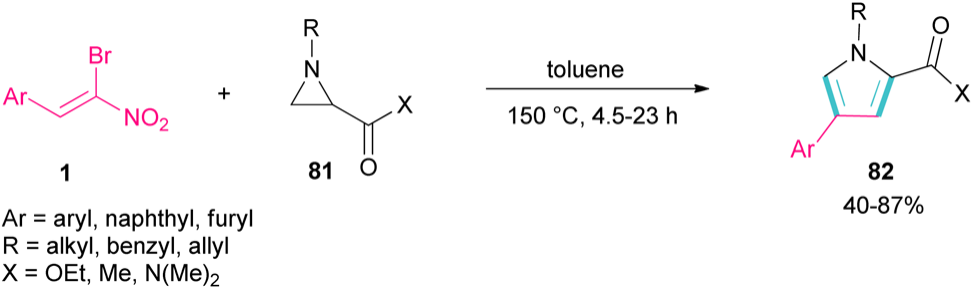
Catalyst-free reaction of unactivated aziridines with β-bromo-β-nitrostyrenes.

**Scheme 35:**
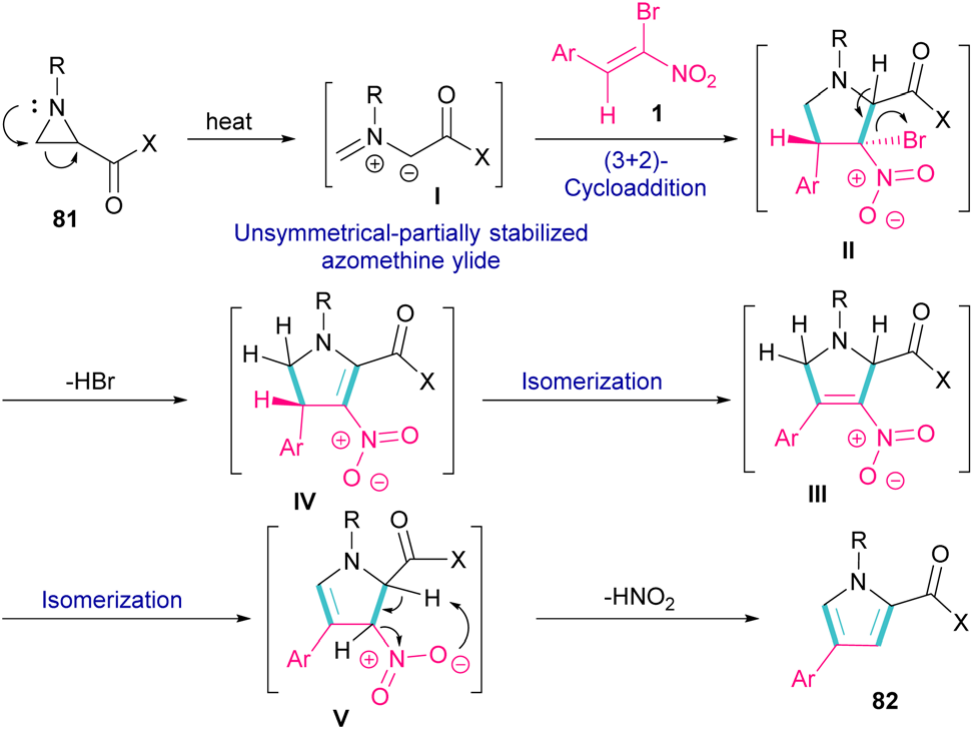
Plausible mechanism for reaction of unactivated aziridines with β-bromo-β-nitrostyrenes.

Another domino reaction was carried out by the Khan research team to construct 1,2,4-trisubstituted pyrrole derivatives 82 from 1,3-dipolar cycloaddition of unactivated aziridines 81 with β-bromo-β-nitrostyrenes 1 (Scheme 34).^63^ This reaction involved *in situ* generated unsymmetrical azomethine ylide from aziridine, followed by a cascade elimination and aromatization step. As shown in Scheme 35, the mechanism started with simultaneous cleavage of the C–C bond of aziridine 81 under heat to afford azomethine ylide I. The interaction of I with β-bromo-β-nitrostyrene 1 resulted in the unstable cycloadduct II, which readily underwent E2 elimination of HBr to render III. In this step, III was isomerized to IV and then V, followed by the elimination of HNO_2_ to form product 82.

**Scheme 36:**
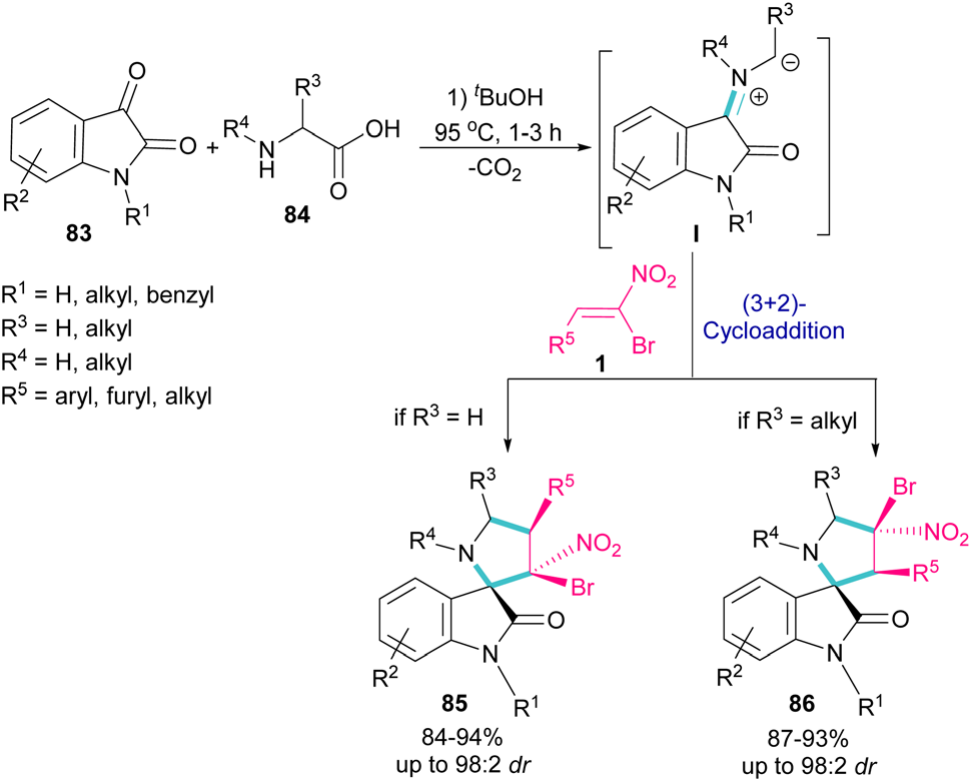
Reaction of oxindole, bromonitrostyrenes and α-amino acids.

In 2019, Ganesh and co-workers developed a multi-component reaction, including oxindoles 83, bromonitrostyrenes 1 and α-amino acids 84 to generate tetra-substituted α-spiropyrrolidine structures 85 and 86 (Scheme 36).^64^ In the first step, azomethine ylide I was formed from the condensation of oxindole and α-amino acid. Next, (3 + 2)-cycloaddition of bromonitrostyrene with I provided spiropyrrolidine. Two diastereomers could be obtained depending on the substituent at the α-position of amino acid. If R^3^ = H, diastereomer 85 was the major product, while spiropyrrolidine 86 was obtained as a major diastereomer when R^3^ = alkyl. The diastereoselectivity may be due to the steric effects in transition states of azometine ylides.

## 3. Conclusions

In this review, we have described the advances in the synthesis of carbocyclic and heterocyclic compounds from the reaction of α-bromonitrostyrenes with various coupling reactants in straightforward and atom economical manners. Organocatalytic domino Michael reactions offer a direct and sustainable route for the synthesis of diastereo- and enantioselective bioactive products under mild reaction conditions. Although it seems that the use of Lewis acid metal catalysts and bases as promoters in the reactions of α-bromonitrostyrenes could be studied further. Considering the great potential of α-bromonitrostyrene as a reactive dipolarophile with good leaving groups (bromo and nitro), the use of this synthon in the cross-coupling reactions, as well as regio- and stereoselective syntheses can provide useful insights for further researches in this field. In our opinion, α-bromonitrostyrenes can be considered as a simple and accessible building block for the construction of more complex organic molecules.

## Conflicts of interest

There are no conflicts to declare.

## Supplementary Material
